# Effect of Herbal and Fluoride Mouth Rinses on* Streptococcus mutans* and Dental Caries among 12–15-Year-Old School Children: A Randomized Controlled Trial

**DOI:** 10.1155/2017/5654373

**Published:** 2017-03-02

**Authors:** Vinej Somaraj, Rekha P. Shenoy, Ganesh Shenoy Panchmal, Vijaya Kumar, Praveen S. Jodalli, Laxminarayan Sonde

**Affiliations:** ^1^Department of Public Health Dentistry, Rajas Dental College & Hospital, Tirunelveli, Tamil Nadu, India; ^2^Department of Public Health Dentistry, Yenepoya Dental College, Mangalore, Karnataka, India; ^3^Department of Periodontics, Yenepoya Dental College, Mangalore, Karnataka, India

## Abstract

To assess and compare the effect of herbal and fluoride mouth rinses on* Streptococcus mutans *count and glucan synthesis by* Streptococcus mutans* and dental caries, a parallel group placebo controlled randomized trial was conducted among 240 schoolchildren (12–15 years old). Participants were randomly divided and allocated into Group I (0.2% fluoride group), Group II (herbal group), and Group III (placebo group). All received 10 ml of respective mouth rinses every fortnight for a period of one year. Intergroup and intragroup comparison were done for* Streptococcus mutans* count and glucan synthesis by* Streptococcus mutans* and dental caries.* Streptococcus mutans* count showed a statistically significant difference between Group I and Group III (*p* = 0.035) and also between Group II and Group III (*p* = 0.039). Glucan concentration levels showed a statistically significant difference (*p* = 0.024) between Group II and Group III at 12th month. Mean DMF scores showed no statistical difference between the three groups (*p* = 0.139). No difference in the level of significance was seen in the intention-to-treat and per-protocol analysis. The present study showed that both herbal and fluoride mouth rinses, when used fortnightly, were equally effective and could be recommended for use in school-based health education program to control dental caries. Trial registration number is CTRI/2015/08/006070.

## 1. Introduction

Dental caries is the most prevalent, modern, lifestyle-dependent disease of humans which is a continuum resulting from many cycles of demineralization and remineralization [1, 2]. Dental caries is progressive and cumulative in nature and becomes more complex over time. Pain from carious teeth can compromise children's quality of life as concentration and participation in school are affected, thereby not only hampering their play and development but also denying them the full benefit of schooling. Disparities in oral health status and in the use of services exist for population groups at all ages [3].

Fluoride is considered a strategy to control caries at either the community or individual levels. Modes of fluoride application include the following: water fluoridation, fluoride dentifrice, fluoride rinse, professional fluoride application, and fluoride-releasing dental materials. Assuring an adequate fluoride exposure is at the heart of numerous school-based activities aiming at improving children's oral health status [3]. Scientific evidence exists on the caries management through effective therapeutic interventions like fluoride [4–8], but a little is known about long-term effects of herbal products. Herbal extracts like chamomile,* Ocimum*, and* Echinacea* when used topically provide therapeutic benefits in the oral cavity [9–13]. Despite the added benefits provided by herbal products when compared to chemical mouth rinses such as greater compliance, cost-effectiveness, nontoxic nature, and proven antibacterial properties, there has been no study reported in literature that has compared the effects of fluoride and herbal products for the prevention of dental caries especially in a school setting on a long-term basis.

The aim of the present study was to assess and compare whether there was any variation in* Streptococcus mutans* counts and the level of glucan synthesis by* Streptococcus mutans* and dental caries among school children who received herbal and fluoride mouth rinses.

## 2. Materials and Methods

A randomized controlled trial (parallel group, placebo controlled) was conducted (December, 2014–December, 2015) to assess and compare the effects of herbal and fluoride mouth rinses on* Streptococcus mutans* count and glucan synthesis by* Streptococcus mutans* and dental caries among 12–15-year-old school children in Mangalore, Karnataka, India. The protocol for the study was presented before University Ethics Committee and request for ethical clearance was made. Permission to conduct the study was obtained from the Block Educational Officer, Mangalore Taluk (South), Karnataka, India. Permission was also obtained from concerned school authorities. All procedures carried out in the study were in full accordance with the World Medical Association Declaration of Helsinki. Assent form was duly signed from each study participant and informed consent was obtained from the parents of the study participants.

The sample size was determined using nMaster 2.0 Sample Size Software based means obtained from a previous study [6]. The calculated sample size (minimum) was 70 per group and to compensate for possible attrition an additional 5 subjects (7%) were allocated to all the three groups, respectively. A total of 256 schoolchildren were approached from four schools of Mangalore, Karnataka, India, of which 240 met the inclusion (consenting to participate and being caries-free) and exclusion criteria (participants with any history of allergy; participants under any medications such as anti-inflammatory drugs, antibiotics, and steroids; participants with any pathological conditions requiring emergency treatment; participants with moderate-to-severe gingival or periodontal pathologies; participants who have already undergone any type of topical fluoride application).

Random sequence was generated online to randomize the 240 schoolchildren using Stratified Block Randomization 40 equal blocks (6 in each block) with different ages to define the different strata. To conceal the allocation of study participants Sequentially Numbered Opaque Sealed Envelope (SNOSE) was used. The primary investigator was involved in the random allocation sequence and enrolment of participants to the assigned interventions. Prior to the study the principal investigator was trained and calibrated with other investigators to record the caries status according to the International Caries Detection and Assessment System (ICDAS) in the Department of Public Health Dentistry, Yenepoya Dental College, Mangalore, India.

The examination was done by a single examiner with assistance from a trained recorder in schools during working days. Group 1 received fluoride mouth rinse (0.2% sodium fluoride), Group 2 received herbal mouth rinse (Freshol), and Group 3 received a placebo (mint flavour added in distilled water) mouth rinse. All mouth rinses were rinsed for 1 minute, fortnightly over a period of 1 year. All the mouth rinses were alcohol-free and freshly prepared on the day of intervention at the Yenepoya Research Centre using triple distilled water as a base after obtaining permission from the Deputy Director, Yenepoya Research Centre. Freshol (herbal mouthwash) was procured from the pharmaceutical company (Father Muller Charitable Institution, Mangalore, Karnataka, India) and diluted as per manufactures instruction. Despite the fact that all mouth rinses were colourless, they were transferred to opaque and numbered plastic bottles to ensure blinding of study participants (single-blind). All mouth rinses had the same flavour (mint flavour was added to all). The investigator visited the schools every fortnight for a period of 1 year. All students were assembled together and instructed to use the mouth rinse (10 ml to be rinsed for 1 minute) in front of the investigator. Students were advised to refrain from eating and gargling the mouth for half an hour after the use of mouth rinse. Samples of unstimulated saliva (3 ml) were collected at Baseline, 6th month, and 12th month, from which* Streptococcus mutans* were identified, counted, and recorded [14]. From the isolates of* Streptococcus* mutans, glucan synthesis was studied by phenol sulphuric acid method at Baseline, 6th month, and 12th month [15]. Caries assessment of study participants was done using International Caries Detection and Assessment System (ICDAS) criteria for detection of caries on coronal tooth surfaces [16–18].

**Figure 1:**
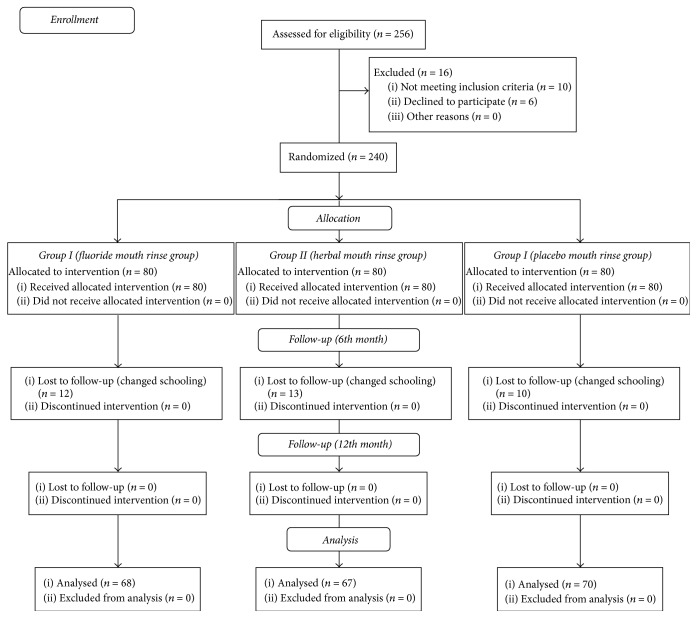
Flow diagram of study protocol.

Descriptive statistics (mean, standard deviation, and frequency distribution) were obtained from the data. Intergroup comparison of* Streptococcus mutans* count and glucan concentration levels at Baseline, 6th month, and 12th month was done using Analysis of Variance (ANOVA). Pair-wise intergroup comparison of* Streptococcus mutans* count and glucan concentration levels at Baseline, 6th month, and 12th month was done using Analysis of Variance (ANOVA) after adjustment for multiple comparisons using Bonferroni Correction. Pair-wise intragroup comparison of* Streptococcus mutans* count and glucan concentration levels in Group I (fluoride mouth rinse), Group II (herbal mouth rinse), and Group III (placebo mouth rinse) at Baseline, 6th month, and 12th month was assessed using General Linear Model for Repeated Measures/Repeated Measures ANOVA (RMA) after adjustment for multiple comparisons using Bonferroni Correction. Intergroup comparison of mean DMF score at Baseline, 6th month, and 12th month was done using Kruskal-Wallis Test. Intragroup comparison of mean DMF score (ICDAS Criteria) in Group I (fluoride mouth rinse), Group II (herbal mouth rinse), and Group III (placebo mouth rinse) at Baseline, 6th month, and 12th month was assessed using Friedman Test. Pair-wise intragroup comparison of mean DMF score (ICDAS Criteria) in Group I (fluoride mouth rinse), Group II (herbal mouth rinse), and Group III (placebo mouth rinse) at Baseline, 6th month, and 12th month was assessed using Wilcoxon Signed Ranks Test. All statistical tests were done using SPSS 21.0 (Statistical Package for Social Sciences; IBM Statistics, 2012). Statistical significance was set at 5%. The study protocol is depicted as a flow diagram (Figure 1)

## 3. Results

The mean age of study participants was 13.86 ± 0.85. Out of the total 240 eligible study participants, 155 (64.6%) were males and 85 (35.4%) were females. Upon completion of the study only 205 participants remained, and 35 (14.58%) were lost to follow-up. Thus intention-to-treat and per-protocol analysis were performed to find the effects of herbal and fluoride mouth rinses on* Streptococcus mutans* count and glucan synthesis by* Streptococcus mutans* and dental caries.

Per-protocol intergroup comparison of* Streptococcus mutans* count and glucan concentration levels and mean DMF values between Group I (fluoride mouth rinse), Group II (herbal mouth rinse), and Group III (placebo mouth rinse) showed that at Baseline there was no statistical difference between the three groups which substantiated the proper randomization of study thus ensuring that there was no selection bias (Tables 1, 2, and 3).

At 6th month and 12th month, there was statistically significant difference between the three groups with lower* Streptococcus mutans* mean count for Group I and Group II. Pair-wise comparison of* Streptococcus mutans* counts at Baseline, 6th month, and 12th month between Group I (fluoride mouth rinse), Group II (herbal mouth rinse), and Group III (placebo mouth rinse) showed there was statistically significant difference between Group II and Group III at 6th month. At 12th month statistical difference was found between Group I and Group III and between Group II and Group III (Table 1). Intragroup and pair-wise intragroup comparison of* Streptococcus mutans* count at Baseline, 6th month, and the 12th month in Groups I, II, and III showed statistically significant difference with lower mean values at 12th month (Table 4).

Per-protocol intergroup comparison of glucan concentration levels at Baseline, 6th month, and 12th month between Group I (fluoride mouth rinse), Group II (herbal mouth rinse), and Group III (placebo mouth rinse) showed no statistical difference. The pair-wise comparison showed there was statistically significant difference (*p* = 0.042) between Group II and Group III at 12th month (Table 2). Per-protocol intragroup and pair-wise intragroup comparison of glucan concentration levels at Baseline, 6th month, and the 12th month in Group I and Group II showed statistically significant difference with lower mean value at 12th month (Table 5).

The mean DMF score was 1.44 (Baseline); 1.41 (6th month); and 1.44 (12th month). Per-protocol intergroup comparison of mean DMF score at Baseline (*p* = 0.212), 6th month (*p* = 0.404), and 12th month (*p* = 0.349) between Group I (fluoride mouth rinse), Group II (herbal mouth rinse), and Group III (placebo mouth rinse) showed there was no statistical difference between the three groups (Table 3). Per-protocol intragroup comparison of mean DMF score in Groups I, II, and III at Baseline, 6th month, and 12th month showed there was no statistical difference between the three groups. Pair-wise intragroup comparison between Baseline, 6th month, and 12th month also showed there was no statistical difference between the three groups (Table 6).

There was no difference between intention-to-treat and per-protocol analysis.

## 4. Discussion

Dental caries is considered as the most prevalent infectious oral disease to afflict mankind. The proportion of the world's population affected by dental caries increased dramatically once refined carbohydrates became available to those within developed and developing countries. The caries process is dependent upon the interaction of protective and deleterious factors. Demineralization and remineralization of enamel are continuous processes that are intimately related [19, 20].

Fluorides play a key role in the prevention and control of dental caries. An important landmark in the history of dentistry is the discovery of the anticariogenic properties of fluoride [21]. Substantial evidence is there in the literature that validated the effectiveness of fluoride mouth rinses on dental caries among schoolchildren [22–28]. Herbs with medicinal properties are a useful and effective source for treatment of various disease processes. Herbal alternatives are easily available and cost-effective and have increased shelf life, low toxicity, and lack of microbial resistance. Herbal agents have a multitude of uses in dentistry as antimicrobial plaque agents, antifungals, and antibacterials and are used in gingivitis and periodontitis to improve immunity [29]. In a study conducted by Mehta et al. [13], comparison of the efficacy of a commercially available homeopathic mouthwash (Freshol) was done with chlorhexidine on plaque status, gingival status, and salivary* Streptococcus mutans* count which showed that Freshol is better than chlorhexidine in reducing the salivary mutans streptococci count and equieffective to chlorhexidine in altering plaque and gingival scores.

The present study deals with assessment and comparison of herbal and fluoride mouth rinses on dental caries and* Streptococcus mutans* count and glucan synthesis by* Streptococcus* mutans. Schoolchildren were selected in the present study as schools form a platform for students to learn good oral hygiene practices and oral health education programs organized periodically among school going children meet their demands in treatment needs and promotion. A total of 240 schoolchildren aged between 12 and 15 years were enrolled into the study with a mean age of 13.86 ± 0.85. This range of age group was selected as this group represents a full complement of permanent dentition (except for third molars) and, this time, period represents the end of mixed dentition period. At this range of age the permanent dentition has been exposed to the oral cavity for 3–9 years, so the assessment of caries was found to be more meaningful. In the present study, 64.6% were males and 35.4% females; the reason of higher percentage of males in the present study was that one of the schools recruited for the study was a residential school for boys.

A total of 240 schoolchildren were recruited for the study, of which 35 (14.58%) were lost to follow-up. The minimum sample required for the study was 210 (70 in each group), but, during the sample size calculation, an additional 7% was added to compensate for the attrition of sample population [30]. Even then attrition could not be controlled at the end of 1 year. This finding of attrition rate was less when compared to study done by Moberg Sköld et al. [31] who reported a drop-out of 21% at the end of 3 years. The reason for such high drop-out is due to the change in schools during the promotion to the class grade and duration of the study. As there was high drop-out in the present study, intention-to-treat and per-protocol Analysis were performed to find the effects of herbal and fluoride mouth rinse.

The intention-to-treat and per-protocol analysis showed that at Baseline there was no statistical difference in the* Streptococcus mutans* count, glucan concentration level, and dental caries scores. This validated the randomization process and ensured that there was no selection bias in the recruitment and allocation of study participants to the intervention groups, which can be considered as the strength of the present study.

The intention-to-treat and per-protocol analysis showed the* Streptococcus mutans* count and glucan concentration level were statistically different at 12th month between the three groups (intergroup comparison), with lower mean scores in the group receiving herbal mouth rinse. Post hoc analysis showed that the difference between the group receiving herbal mouth rinse and the one receiving placebo mouth rinse was statistically significant. There was no statistical difference between groups receiving fluoride and placebo mouth rinse and groups receiving fluoride and herbal mouth rinses. The results indicated that herbal mouth rinse was effective in reducing the* Streptococcus mutans* count and glucan synthesis by* Streptococcus mutans*. A similar reduction in* Streptococcus mutans* score was observed in a study conducted by Mehta et al. [13]. Intention-to-treat and per-protocol analysis of intragroup comparison showed that participants receiving fluoride and herbal mouth rinses had an equal reduction in* Streptococcus mutans* count and glucan concentration levels, suggesting that both the interventions were equally effective. A similar reduction in* Streptococcus mutans* count and glucan concentration levels was reported by Umetsu et al. [6] in which only fluoride mouth rinse was compared against nonfluoride rinse users.

The mean difference in DMF score as per ICDAS Criteria among the three groups was statistically insignificant when the intention-to-treat and per-protocol analysis were performed for intergroup comparison among the three groups. This finding is in contradiction with the findings of Umetsu et al. [6] in which the mean DFT scores were statistically lesser for fluoride rinse group.

The fluoride mouth rinsing schedule was done every fortnight as per World Health Organization (WHO) recommendations [32]. As there was no standardized schedule for herbal mouth rinses the same fortnightly intervention was followed. All the mouth rinses were freshly prepared on the day of intervention and the rinsing procedure was under the direct supervision of the principal investigator which ensured that all the study participants had followed the rinsing protocol. No adverse effects for the mouth rinses were reported by subjects to school authorities and in turn to the principal investigator.

The strength of the present study was the study design and the random allocation of subjects to the intervention arms which ensured that there was no selection bias. Other strengths include the intervention being supervised by the principal investigator personally which ensured that all participants were following the proper intervention protocol. As all mouth rinses were water-based and freshly prepared on the day of intervention there were no reports of adverse effects. As the intention-to-treat and per-protocol analysis yielded the same results, it can be concluded that the effect of drop-out was not evident in the present study.

To our knowledge, there is no study reported in the literature which has investigated the effects of herbal and fluoride mouth rinses among schoolchildren. Within the limitations of the present study, it was found that both the formulations were equally effective in reduction of* Streptococcus mutans* count and glucan synthesis by* Streptococcus mutans*.

## 5. Conclusion

Mouth rinsing for the prevention of dental caries in children is well established as a mass prophylactic method and school-based supervised mouth rinses are relatively cost-efficient when compared to other large-scale caries preventive measures. The present study showed the effectiveness of herbal and fluoride mouth rinses in the reduction of* Streptococcus mutans* count and glucan synthesis by* Streptococcus mutans* among schoolchildren who used the mouth rinses for a period of one year. Further long-term clinical trials are recommended to validate the effects of herbal mouth rinses on dental caries.

## Figures and Tables

**Table 1:** Intergroup and pair-wise intergroup comparison of *Streptococcus mutans* count (10^4^ CFU/ml) at Baseline, 6th month, and 12th month (per-protocol analysis).

Time period	Groups	Mean	Standard deviation	*p* value	Pair-wise comparisons (*p* value)		
					Group I-Group II	Group I–Group III	Group II-Group III
Baseline	Group I	2257.69	1198.60	0.086	1.000	0.116	0.263
	Group II	2366.42	1685.54				
	Group III	2830.00	1815.30				

6th month	Group I	2079.23	1053.26	0.032^*∗*^	1.000	0.255	0.031^*∗*^
	Group II	1872.85	1521.57				
	Group III	2519.28	1741.40				

12th month	Group I	1688.46	1026.62	<0.001^*∗*^	0.742	0.020^*∗*^	<0.001^*∗*^
	Group II	1405.00	1355.80				
	Group III	2359.28	1752.31				

Group I = fluoride mouth rinse [*n* = 68],   Group II = herbal mouth rinse [*n* = 67], and Group III = placebo mouth rinse [*n* = 70].

*p* value based on ANOVA Test after adjustment for multiple comparisons using Bonferroni Correction (Post hoc analysis).

^*∗*^Statistically significant (*p* < 0.05).

**Table 2:** Intergroup and pair-wise intergroup comparison of glucan concentration (*μ*g/ml) levels at Baseline, 6th month, and 12th month (per-protocol analysis).

Time period	Groups	Mean	Standard deviation	*p* value	Pair-wise comparisons (*p*-value)		
					Group I-Group II	Group I–Group III	Group II-Group III
Baseline	Group I	36.56	18.74	0.263	0.388	1.000	0.610
	Group II	31.84	16.19				
	Group III	35.72	18.96				

6th month	Group I	35.97	18.63	0.218	0.393	1.000	0.399
	Group II	31.25	16.16				
	Group III	35.86	19.28				

12th month	Group I	32.60	18.71	0.043^*∗*^	0.308	1.000	0.042^*∗*^
	Group II	27.49	16.03				
	Group III	35.06	19.35				

Group I = fluoride mouth rinse [*n* = 68], Group II = herbal mouth rinse [*n* = 67], and Group III = placebo mouth rinse [*n* = 70].

*p* value based on ANOVA Test after adjustment for multiple comparisons using Bonferroni Correction (post hoc analysis).

^*∗*^Statistically significant (*p* < 0.05).

**Table 3:** Intergroup comparison of mean DMF score (ICDAS Criteria) at Baseline, 6th month, and 12th month (per-protocol analysis).

Time period	Groups	*N*	Mean rank	Chi-square value	*p* value
Baseline	Group I	68	102.48	3.099	0.212
	Group II	67	111.46		
	Group III	70	95.02		

6th month	Group I	68	101.23	1.812	0.404
	Group II	67	109.94		
	Group III	70	97.70		

12th month	Group I	68	100.42	1.648	0.349
	Group II	67	109.86		
	Group III	70	98.54		

*p* value based on Kruskal-Wallis Test.

^*∗*^Statistically significant (*p* < 0.05).

**Table 4:** Intragroup and pair-wise intragroup comparison of *Streptococcus mutans* count (10^4^ CFU/ml) in Groups I, II, and III at Baseline, 6th month, and 12th month (Per-protocol analysis).

Time period	*N*	Mean	Standard deviation	*p* value	Pair-wise comparisons (*p* value)		
					Baseline–6th month	Baseline–12th month	6th month–12th month
Group I (fluoride mouth rinse)							
Baseline	68	2312.50	1210.14	<0.001^*∗*^	0.004^*∗*^	<0.001^*∗*^	<0.001^*∗*^
6th month	67	2119.16	1062.10				
12th month	70	1695.83	1043.23				

Group II (herbal mouth rinse)							
Baseline	68	2529.45	1814.17	<0.001^*∗*^	<0.001^*∗*^	<0.001^*∗*^	<0.001^*∗*^
6th month	67	2015.06	1636.66				
12th month	70	1511.64	1429.87				

Group III (placebo mouth rinse)							
Baseline	68	2952.77	1870.80	<0.001^*∗*^	0.001^*∗*^	0.070	<0.001^*∗*^
6th Month	67	2629.86	1786.30				
12th Month	70	2467.36	1801.52				

*p* value based on Repeated Measures ANOVA (RMA) Test after adjustment for multiple comparisons using Bonferroni Correction.

^*∗*^Statistically significant (*p* < 0.05).

**Table 5:** Intragroup and pair-wise intragroup comparison of glucan levels (*μ*g/ml) in Groups I, II, and III at Baseline, 6th month, and 12th month (per-protocol analysis).

Time period	*N*	Mean	Standard deviation	*p* value	Pair-wise comparisons (*p*-value)		
					Baseline–6th month	Baseline–12th month	6th month–12th month
Group I (fluoride mouth rinse)							
Baseline	68	35.61	18.90	<0.001^*∗*^	0.030^*∗*^	<0.001^*∗*^	<0.001^*∗*^
6th month	67	34.96	18.75				
12th month	70	31.31	18.60				

Group II (herbal mouth rinse)							
Baseline	68	32.29	16.30	<0.001^*∗*^	<0.001^*∗*^	<0.001^*∗*^	<0.001^*∗*^
6th month	67	31.68	16.26				
12th month	70	27.89	16.14				

Group III (placebo mouth rinse)							
Baseline	68	36.17	19.26	1.000	0.390	0.061	1.000
6th month	67	36.25	19.50				
12th month	70	35.49	19.63				

*p* value based on Repeated Measures ANOVA (RMA) Test after adjustment for multiple comparisons using Bonferroni Correction.

^*∗*^Statistically significant (*p* < 0.05).

**Table 6:** Intragroup and pair-wise intragroup comparison of mean DMF score (ICDAS Criteria) in Groups I, II, and III (fluoride mouth rinse) at Baseline, 6th month, and 12th month (per-protocol analysis).

Time period (*N* = 68)	Mean	Standard deviation	*p* value Friedman Test	*p* value Wilcoxon Signed Ranks Test		
				Baseline–6th month	Baseline–12th month	6th month–12th month
Group I (fluoride mouth rinse)						
Baseline	1.16	1.42	0.135	0.180	1.000	0.180
6th month	1.10	1.34				
12th month	1.16	1.42				

Group II (herbal mouth rinse)						
Baseline	1.31	1.56	0.174	0.257	0.317	0.102
6th month	1.27	1.54				
12th month	1.32	1.57				

Group III (placebo mouth rinse)						
Baseline	0.95	1.34	0.234	0.426	0.068	0.102
6th month	1.00	1.34				
12th month	1.05	1.32				
